# The role of comorbidity and frailty in shaping the burden of atrial fibrillation: a multinational cross-sectional survey

**DOI:** 10.1038/s41598-026-44800-1

**Published:** 2026-03-30

**Authors:** Adele Ravelli, Caterina Trevisan, José Miguel Rivera-Caravaca, Bruno Micael Zanforlini, Caterina Bosio, Guendalina Graffigna, Giuseppe Sergi, Gheorghe-Andrei Dan, Anca Rodica Dan, Vanessa Roldán, Francisco Marín Ortuno, Søren Paaske Johnsen, Mirko Petrovic, Davide Liborio Vetrano, Donato Giuseppe Leo, Deirdre A. Lane

**Affiliations:** 1 Department of Medicine, University of Padua, Padua, Italy; 2 Aging Research Center, Department of Neurobiology, Care Sciences and Society (NVS), Karolinska Institutet-Stockholm University, Solna, Sweden; 3 Department of Medical Sciences, University of Ferrara, Ferrara, Italy; 4 Faculty of Nursing, University of Murcia, Instituto Murciano de Investigación Biosanitaria Pascual Parrilla (IMIB-Pascual Parrilla), CIBERCV, Murcia, Spain; 5 EngageMinds HUB – Consumer, Food and Health Engagement Research Center, Catholic University of Sacred Heart, Cremona, Italy; 6 Department of Psychology, Catholic University of Sacred Heart, Milan, Italy; 7 Carol Davila University of Medicine, Bucharest, Romania; 8 Academy of the Romanian Scientists, Bucharest, Romania; 9 Cardiology Department, Colentina University Hospital, Bucharest, Romania; 10 Department of Hematology, Hospital Clínico Universitario Virgen de La Arrixaca , University of Murcia, Instituto Murciano de Investigación Biosanitaria Pascual Parrilla (IMIB-Pascual Parrilla), Murcia, Spain; 11 Department of Cardiology, Hospital Clínico Universitario Virgen de La Arrixaca , University of Murcia, Instituto Murciano de Investigación Biosanitaria Pascual Parrilla (IMIB-Pascual Parrilla), CIBERCV, Murcia, Spain; 12 Aalborg University Hospital, Aalborg, Denmark; 13 Danish Center for Health Services Research, Department of Clinical Medicine, Aalborg University, Aalborg, Denmark; 14 Section of Geriatrics, Department of Internal Medicine and Paediatrics, Ghent University, Ghent, Belgium; 15 Stockholm Gerontology Research Center, Stockholm, Sweden; 16 Liverpool Centre for Cardiovascular Sciences, University of Liverpool and Liverpool Heart and Chest Hospital, Liverpool, UK; 17 Department of Cardiovascular and Metabolic Medicine, Faculty of Health and Life Sciences, Institute of Life Course and Medical Sciences, University of Liverpool, Liverpool, UK; 18 Aalborg Universitet, Aalborg, Denmark; 19 The University of Liverpool, Liverpool, UK; 20 Universiteit Gent, Gent, Belgium; 21 Karolinska Institutet, Stockholm, Sweden; 22 Consiglio Nazionale delle Ricerche, Rome, Italy; 23 Universitatea de Medicina si Farmacie “Carol Davila” din Bucuresti, Bucharest, Romania; 24 Università degli Studi di Padova, Padua, Italy; 25 Faculty of Medicine, University of Belgrade, Belgrade, Serbia; 26 Moverim Consulting sprl, Brussels, Belgium; 27 Advice Pharma Group srl, Milan, Italy; 28 Ontonix, Como, Italy; 29 Arrhythmia Alliance, Chipping Norton, UK; 30 European Union Geriatric Medicine Society AISBL, Brussels, Belgium; 31 Istituto Superiore di Sanità, Rome, Italy; 32 Università Cattolica del Sacro Cuore, Milan, Italy; 33 Heart Care Foundation Onlus, Florence, Italy; 34 The European Institute for Innovation Through Health Data, Gent, Belgium; 35 The University of Manchester, Manchester, UK; 36 Universidad de Murcia, Murcia, Spain; 37 Medical University Plovdiv, Plovdiv, Bulgaria

**Keywords:** Atrial fibrillation, Older adults, Frailty, Comorbidities, Quality of life, Health outcomes, Cardiology, Diseases, Health care, Medical research

## Abstract

Atrial fibrillation (AF) is the most common arrhythmia in older adults and often coexists with other chronic conditions, exacerbating physical and cognitive decline and contributing to frailty. The interplay between frailty and comorbidity in AF remains underexplored, particularly regarding quality of life (QoL), health management, and outcome prioritization. Within the AFFIRMO project, this study investigated the experiences of older adults with AF and at least one chronic condition via an online survey. Frailty was assessed using the FRAIL questionnaire, and participants were grouped by frailty status and number of comorbidities. Health-related quality of life (HRQoL) was measured using the EQ-5D-3L and Visual Analogue Scale (VAS). Challenges in health management and prioritized outcomes were also explored. We included 659 participants (median age 72 years, 52.8% female). Those with pre-frailty or frailty and ≥ 3 comorbidities reported the poorest HRQoL. Comorbidity, particularly combined with frailty, was associated with health management difficulties, including healthcare visits, polypharmacy, and mobility limitations. Across all groups, maintaining independence and improving QoL were prioritized outcomes. Pain relief was especially important for those with higher comorbidities. In older adults with AF, comorbidity and frailty significantly affect QoL and health burden. Tailored, patient-centred care strategies and routine assessment of frailty and comorbidity are essential to improve care coordination and outcomes.

**Supplementary Information:**

The online version contains supplementary material available at 10.1038/s41598-026-44800-1.

## Introduction

Atrial fibrillation (AF) is the most common arrhythmia worldwide, with its prevalence rising sharply with advancing age^1^. This condition imposes a significant burden on patients, caregivers, and healthcare systems, which is further compounded in an aging population^2^. In older adults, AF is a major driver of chronic and acute conditions, including stroke, heart failure, and chronic kidney disease^3^, often resulting in comorbidity, which, in turn, amplifies the risk of adverse health outcomes such as hospitalisations, falls, and mortality^4^.

Comorbidity, defined as the coexistence of two or more chronic conditions, exacerbates physical and cognitive decline, ultimately leading to functional impairment. This arises from the interplay between diseases, which undermines compensatory mechanisms and resilience, as well as polypharmacy, which heightens the risks of drug–drug and drug–disease interactions, as well as inappropriate medication use^5^. These challenges are further intensified by the fragmented nature of care, resulting in suboptimal management and a heavy treatment burden, with patients facing numerous medical appointments with often poor coordination among specialists^6^.

Another complex challenge in managing older adults with AF is frailty, a state of reduced physiological reserve, autonomy and enhanced vulnerability to stressors^7^. AF independently increases the risk of frailty, regardless of age, sex, or comorbidities^8^. Moreover, a bidirectional relationship has been hypothesized, where frailty increases the risk of AF and vice versa^9^. Frailty frequently coexists with comorbidity, as they share overlapping pathophysiological mechanisms and risk factors^10^. However, while more than two-thirds of older adults with frailty have comorbidity, less than one-fifth of those with comorbidity are frail^11^. This distinction underscores the importance of assessing frailty in older adults with AF and comorbidity, as its presence -or absence- may influence clinical decision-making and management strategies. Indeed, within this intricate interplay, the complexity of managing older adults with AF extends beyond disease control. The coexistence of multiple conditions profoundly impacts health-related quality of life (HRQoL) not only through additive effects but also via synergistic or antagonistic interactions that further exacerbate the burden on individuals^12^. Frailty adds to this challenge, being associated with impaired mobility, social isolation, loneliness, and depression, all of which significantly contribute to a decline in quality of life (QoL)^13,14^.

For these reasons, the care of older adults with AF should move beyond a solely disease-centred perspective, by carefully addressing comorbidity and frailty together while prioritising patient-relevant outcomes, health challenges, and QoL alongside traditional clinical targets^15^. Despite their importance, frailty and comorbidity are not routinely assessed in clinical practice, and no study to date has explored the differential impact of these conditions on shaping the QoL and care needs of patients with AF. To address this gap, this study aims to investigate the interplay between frailty and the number of comorbidities in influencing older adults with AF in terms of QoL, primary challenges in daily health management, and prioritized health outcomes.

## Methods

This analysis is part of the “Atrial fibrillation integrated approach in frail, multimorbid, and polymedicated older people” (AFFIRMO) project^16^. One of the objectives of the AFFIRMO project was to evaluate the experiences of patients living with AF and multimorbidity, and to identify their needs and the main aspects they consider as quality indicators of care. Specifically, an online survey was developed by a multidisciplinary team of physicians and researchers skilled in AF, geriatricians, psychologists, and patient representatives. The survey was translated from English to Italian, Romanian, Spanish, and Danish and distributed on-line and disseminated by researchers at outpatient and inpatient clinics and a patient organisation, from 31 May 2022 to 31 January 2023^17^. To be eligible for inclusion criteria for participation in the study, patients had to be ≥ 18 years old, a diagnosis of AF, at least one additional comorbid condition, and able to provide informed consent.

The study protocol received approval from the ethical committees of all participating countries: the United Kingdom (REC 21/YH/0307), Italy (015534, ref. 5308/AO/22), Spain (2021-12-15-HCUVA), and Romania (2SNI/13.01.2022). In accordance with the guidelines of the Danish Research Ethics Committee, ethical approval was not necessary for Denmark. Before commencing the online survey, each participant was required to provide informed consent to participate in the study.

### Survey content

This survey collected information on sociodemographic characteristics, medical history, problems in managing health and health outcomes of importance.

Specifically, patients were asked to identify the main problems encountered in managing their health and the health outcomes they prioritized from a broad range of issues, carefully designed by the multidisciplinary team to encompass various aspects of health management and priorities. A comprehensive description of the information collected is provided in Supplementary Table S1 online.

Additionally, the survey included the following validated questionnaires to assess frailty and HRQoL:

- FRAIL questionnaire^18^: 5-question assessment tool to screen for frailty in older adults in the following domains: fatigue, resistance (ability to climb one flight of stairs), aerobic capacity (ability to walk one block), illnesses (presence of > 5 illnesses), and loss of weight (> 5% in the past 6 months).
- EuroQol-5 Dimension, 3 level version (EQ-5D-3L)^19^: evaluates five dimensions of health status: Mobility, Self-care, Usual activities, Pain/discomfort, and Anxiety/depression. Each dimension includes three levels of severity: no problems (level 1), moderate problems (level 2), and extreme problems (level 3). A global index score closer to 1 denotes a better perception of one’s health status (18). The Visual Analogue Scale (VAS) asks respondents to rate their overall health on a scale from 0 (the worst imaginable health) to 100 (the best imaginable health).

### Main exposures

Comorbidity burden was operationalised as a simple disease count, with each condition weighted equally. Patients with AF were categorized into two groups based on the median number of comorbidities observed in our sample (median = 3). The threshold of ≥ 3 comorbidities was selected as a data-driven cut-off reflecting the distribution of comorbidities in our sample and allowing identification of individuals with a higher cumulative disease burden.

Based on the scores from the FRAIL questionnaire, patients were classified as non-frail (score = 0), pre-frail (score = 1–2), and frail (score = 3–5). Pre-frailty is conceptualised as an intermediate, potentially reversible stage in the frailty spectrum, characterised by the presence of one or two frailty criteria according to Fried’s phenotype model. It reflects a state of increased vulnerability to stressors compared to robust individuals, but without the full clinical manifestation of frailty^7^.

Patients were then further grouped into one of six groups based on the interaction between comorbidity and frailty:

Group 1: No frailty, < 3 comorbidities; Group 2: No frailty, ≥ 3 comorbidities; Group 3: Pre frailty, < 3 comorbidities; Group 4: Pre frailty, ≥ 3 comorbidities; Group 5: Frailty, < 3 comorbidities; Group 6: Frailty, ≥ 3 comorbidities.

### Outcomes

The primary outcomes considered were HRQoL, the main problems patients encountered in managing their health, and the health outcomes they prioritized, as described above.

### Statistical analysis

The distribution of quantitative variables was assessed for normality using the Shapiro–Wilk and Kolmogorov–Smirnov tests, together with graphical methods (histograms and Q–Q plots). All quantitative variables were not normally distributed, therefore they are expressed as median (interquartile range, IQR). Categorical variables are presented as absolute frequencies and percentages. Comparisons between characteristics of participants assigned to the six different groups based on comorbidity and frailty status was conducted using the Kruskal–Wallis for quantitative variables or Chi-squared test for categorical variables.

To investigate the association between the different groups (considering Group 1 as the reference) and the total scores on the EQ-5D-3L and VAS, generalized linear regression models were employed. Unadjusted models and models adjusted a priori for age, sex, level of education, and living arrangements were performed, as these variables were considered plausible confounders based on their established associations with frailty, comorbidity, and HRQoL. In addition, binary logistic regression models were used to explore whether the different groups could predict the presence of moderate or extreme problems in the EQ-5D domains. Binary logistic regression models were also employed to verify whether the problems and health outcomes perceived as most important by AF patients could be associated with their comorbidity/frailty status. All analyses were conducted with IBM Statistical Package for the Social Sciences (SPSS) version 29^20^, with *p*-values < 0.05 considered statistically significant.

## Results

The sample consisted of 659 individuals, median age 72 years (IQR 65-77) and 348 (52.8%) females. Table 1 shows the demographic and clinical characteristics overall and in the six groups by frailty and comorbidity status. Most patients had a high level of education, led an independent life, with most living at home with their families (63.6%). Only a small minority required part-time or full-time assistance (7.6% and 3.3%, respectively). Hypertension (59.0%) and coronary heart disease (60.5%) were the most frequently observed comorbidities. Overall, 25.6% of patients were classified as non-frail, 55.4% as pre-frail, and 19.0% as frail. Regarding comorbidity, 55.1% of the sample had three or more chronic conditions. When considering the combination of frailty and comorbidity status, 85 (12.9%) individuals were non-frail with < 3 chronic conditions (Group 1), 84 (12.7%) were non-frail but had ≥ 3 comorbidities (Group 2), 167 (25.3%) were pre-frail with < 3 chronic conditions(Group 3), 198 (30.0%) were pre-frail and had ≥ 3 comorbidities (Group 4), 44 (6.7%) were frail but with < 3 chronic conditions (Group 5) and 81 (12.3%) were frail and had ≥ 3 comorbidities (Group 6). The six groups exhibited significant differences in age, level of education and living arrangements, but no sex differences (Table 1). Groups 1 and 3 were younger compared to the other groups. Group 6 had the highest frequency of individuals with low education (25.9%), while Groups 1, 3, and 5 had the highest proportion of individuals with a degree or above (48.2%, 55.7%, and 47.7%, respectively). Individuals with ≥ 3 comorbidities (Groups 2, 4, and 6) showed a greater need for assistance at home (*p* < 0.001).

**Table 1:** Characteristics of patients with atrial fibrillation participating in the survey categorized by frailty and multimorbidity.

N (%)	Overall	Multimorbidity/frailty groups						*p*-value
		Group 1 No frailty, < 3 comorbidities	Group 2 No frailty, ≥ 3 comorbidities	Group 3 Pre-frailty, < 3 comorbidities	Group 4 Pre-frailty, ≥ 3 comorbidities	Group 5 Frailty, < 3 comorbidities	Group 6 Frailty, ≥ 3 comorbidities	
n	659	85 (12.9)	84 (12.7)	167 (25.3)	198 (30.0)	44 (6.7)	81 (12.3)	
Age (years)	72 [65–77]	69 [62–76]^b,d,f^	74 [68–79]^a,c^	69 [62–76]^b,d,f^	74 [67–78]^a,c^	72 [65–75]	75 [68–79]^a,c^	< 0.001
Female sex	348 (52.8)	45 (52.9)	47 (56.0)	80 (47.9)	111 (56.1)	23 (52.3)	42 (51.9)	0.728
Level of education*								< 0.001
Primary	94 (14.3)	7 (8.2)^f^	14 (16.7)	18 (10.8)^d,f^	33 (16.7)^c^	1 (2.3)^f^	21 (25.9)^a,c,e^	
Secondary	265 (40.2)	35 (41.2)	36 (42.9)	52 (31.1)	90 (45.5)	19 (43.2)	33 (40.7)	
Degree or above	281 (42.6)	41 (48.2)	32 (38.1)	93 (55.7)	69 (34.8)	21 (47.7)	25 (30.9)	
Other	19 (2.9)	2 (2.4)	2 (2.4)	4 (2.4)	6 (3.0)	3 (6.8)	2 (2.5)	
Living arrangement*								< 0.001
At home alone	168 (25.5)	30 (35.3)^d,f^	24 (28.6)^c^	37 (22.2)^b,d,f^	46 (23.2)^a,c^	10 (22.7)	21 (25.9)^a,c^	
At home with family	419 (63.6)	53 (62.4)	49 (58.3)	123 (73.7)	117 (59.1)	31 (70.5)	46 (56.8)	
At home with part-time assistance	50 (7.6)	2 (2.4)	9 (10.7)	5 (3.0)	26 (13.1)	1 (2.3)	7 (8.6)	
At home with full-time assistance or in NH	22 (3.3)	0	2 (2.4)	2 (1.2)	9 (4.5)	2 (4.5)	7 (8.6)	
Marital status								0.273
Married/partnered	448 (68.0)	59 (69.4)	54 (64.3)	120 (71.9)	128 (64.6)	34 (77.3)	53 (65.4)	
Separated/divorced	61 (9.3)	6 (7.1)	8 (9.5)	19 (1.4)	19 (9.6)	2 (4.5)	7 (8.6)	
Single/never married	39 (5.9)	7 (8.2)	6 (7.1)	12 (7.2)	8 (4.0)	3 (6.8)	3 (3.7)	
Widowed	111 (16.8)	13 (15.3)	16 (19.0)	16 (9.6)	43 (21.7)	5 (11.4)	18 (22.2)	
Smoking habits								0.06
Current smoker	33 (5.0)	3 (3.5)	6 (7.1)	6 (3.6)	6 (3.0)	7 (15.9)	5 (6.2)	
Former smoker	281 (42.6)	35 (41.2)	39 (46.4)	74 (44.3)	83 (41.9)	13 (29.5)	37 (45.7)	
Never smoker	345 (52.4)	47 (55.3)	39 (46.4)	87 (52.1)	109 (55.1)	24 (54.5)	39 (48.1)	
Comorbidities								
Hypertension	389 (59.0)	28 (32.9)^b,d,f^	68 (81.0)^a,c^	62 (37.1)^b,d,f^	151 (76.3)^a,c^	25 (56.8)	55 (67.9)^a,c^	< 0.001
Coronary heart disease	399 (60.5)	31 (36.5)^b,d,f^	67 (79.8)^a,c,e^	74 (44.3) ^b,d,f^	147 (74.2) ^a,c,e^	19 (43.2) ^b,d,f^	61 (75.3) ^a,c,e^	< 0.001
Previous stroke	57 (8.6)	3 (3.5)	10 (11.9)	5 (3.0)^d^	27 (13.6)^c^	1 (2.3)	11 (13.6)	< 0.001
Diabetes	108 (16.4)	7 (8.2)^b,d^	23 (27.4)^a,c,e^	7 (4.2)^b,d,f^	53 (26.8) ^a,c,e^	1 (2.3) ^b,d^	17 (21.0)^c^	< 0.001
Thyroid disease	108 (16.4)	6 (7.1)^d,f^	16 (19.0)	16 (9.6) ^d,f^	47 (23.7)^a,c,e^	2 (4.5)^d,f^	21 (25.9)^a,c,e^	< 0.001
COPD	41 (6.2)	1 (1.2)^b^	12 (14.3)^a,c^	1 (0.6)^b,d^	19 (9.6)^c^	1 (2.3)	7 (8.6)	< 0.001
Gastrointestinal diseases	128 (19.4)	7 (8.2) ^d,f^	19 (22.6)	15 (9.0) ^d,f^	63 (31.8)^a,c,e^	2 (4.5) ^d,f^	22 (27.2) ^a,c,e^	< 0.001
Chronic liver disease	19 (2.9)	0	4 (4.8%)	0f.	9 (4.5)	0	6 (7.4)^f^	0.003
Chronic kidney disease	62 (9.4)	1 (1.2)^d,f^	10 (11.9)	3 (1.8)^d,f^	32 (16.2)^a,c,e^	0^d,f^	16 (19.8)^a,c,e^	< 0.001
Parkinson’s Disease	8 (1.2)	0	0	2 (1.2)	5 (2.5)	0	1 (1.2)	0.363
Multiple Sclerosis	3 (0.5)	0	0	0	2 (1.0)	0	1 (1.2)	0.525
Cognitive disturbances	48 (7.3)	3 (3.5)	9 (10.7)	3 (1.8)^d^	21 (10.6)^c^	2 (4.5)	10 (12.3)	0.004
Osteoarthritis	165 (25.0)	13 (15.3)^b,d^	31 (36.9)^a,c,e^	17 (10.2)^b,d,f^	72 (36.4)^a,c,e^	5 (11.4)^b,d^	27 (33.3)^c^	< 0.001
Osteoporosis	51 (7.7)	1 (1.2)^d^	11 (13.1)	5 (3.0)^d^	25 (12.6)^a,c^	0	9 (11.1)	< 0.001
Rheumatoid arthritis	29 (4.4)	3 (3.5)	3 (3.6)	2 (1.2)^d^	15 (7.6)^c^	0	6 (7.4)	0.025
Chronic pain	77 (11.7)	2 (2.4)^d,f^	13 (15.5)^c^	4 (2.4)^b,d^	39 (19.7)^a,c^	3 (6.8)	16 (19.8)^a,c^	< 0.001
Cancer	40 (6.1)	2 (2.4)^d^	2 (2.4)^d^	5 (3.0)^d^	23 (11.6)^a,b,c^	2 (4.5)	6 (7.4)	0.003
Hearing problems	105 (15.9)	7 (8.2)^d,f^	18 (21.4)^c^	8 (4.8)^b,d,f^	46 (23.2)^a,c^	4 (9.1)	22 (27.2)^a,c^	< 0.001
Vision problems	119 (18.1)	5 (5.9)^b,d,f^	23 (27.4)^a,c,e^	11 (6.6)^b,d,f^	54 (27.3)^a,c,e^	0^b,d,f^	26 (32.1)^a,c,e^	< 0.001
EQ-5D								
EQ-5D total	0.64 [0.38–0.79]	0.79^b,d,f^ [0.53–1.00]	0.64^a,c^ [0.38–0.79]	0.73^b,d,f^ [0.53–0.85]	0.53^a,c^ [0.19–0.73]	0.61 [0.40–0.87]	0.53^a,c^ [0.34–0.85]	< 0.001
EQ-5D VAS	60 [50–78.7]	75^b,d,f^ [60–88.5]	60^a^ [50–78.8]	75^d,f^ [60–85]	60^a,c^ [50–75]	62.5 [50–80]	60^a,c^ [50–75]	< 0.001

Numbers are median [interquartile range] or number (%), as appropriate. Post-hoc comparisons: ^a^significantly different (*p* < 0.05) versus Group 1 (reference); ^b^significantly different versus Group 2; ^c^significantly different versus Group 3; ^d^significantly different versus Group 4; ^e^significant difference versus Group 5; ^f^significant differen versus Group 6. *Post-hoc comparisons refers to significant differences for the main variable. Group 1 includes non-frail individuals with < 3 comorbidities, Group 2 includes non-frail individuals with ≥ 3 comorbidities, Group 3 includes pre-frail individuals with < 3 comorbidities, Group 4 includes pre-frail individuals with ≥ 3 comorbidities, Group 5 includes frail individuals with < 3 comorbidities, Group 6 includes frail individuals with ≥ 3 comorbidities.

COPD, Chronic obstructive pulmonary disease; EQ-5D, EuroQoL-5 Dimension; NH, nursing home; VAS = Visual Analogue Scale.

### Health-related quality of life

There were significant differences in health perception among the groups. Specifically, individuals with no frailty or pre-frailty and < 3 comorbidities perceived a better overall HRQoL (EQ-5D-3L total score, VAS score) (Table 1) compared to all other groups. In contrast, those living with pre-frailty or frailty and who had ≥ 3 comorbidities reported the worst HRQoL.

Results from linear regression analysis suggested a negative association between frailty/comorbidity groups and the total EQ-5D-3L and VAS score. Compared to Group 1, having ≥ 3 comorbidities or frailty (all groups except Group 3) was associated with a lower HRQoL in both unadjusted and adjusted analyses (Table 2). Notably, a similar pattern of HRQoL reduction was observed in individuals who were non-frail but had ≥ 3 comorbidities (Group 2) and those who were frail but had < 3 comorbidities (Group 5). This pattern differed from that observed in groups with the combination of both conditions (pre-frailty or frailty and multiple comorbidities), who exhibited the most pronounced impairments.

**Table 2:** Linear regression analysis for the association between comorbidity/frailty groups, EQ-5D total scores and VAS in patients with atrial fibrillation.

Variable	β coefficient (95%CI), p-value			
	EQ-5D VAS		EQ-5D total	
	Unadjusted	Adjusted	Unadjusted	Adjusted
Group 1 No frailty, < 3 comorbidities	Ref	Ref	Ref	Ref
Group 2 No frailty, 3 + comorbidities	**− 10.80 (− 16.70; − 4.89), *p* < 0.001**	**− 9.50 (− 15.33; − 3.67), *p* = 0.001**	**− 0.22 (− 0.38; − 0.11),** ***p* < 0.001**	**− 0.18 (− 0.29; − 0.06),** ***p* = 0.002**
Group 3 Pre-frailty, < 3 comorbidities	**− **3.50 (**− **8.61; 1.61), *p* = 0.18	**− **3.74 (**− **8.75;1.26), *p* = 0.14	**− **0.04 (**− **0.14;0.06), *p* = 0.45	**− **0.04 (**− **0.13;0.06), *p* = 0.42
Group 4 Pre-frailty, 3 + comorbidities	**− 14.10 (− 19.08; -9.12), *p* < 0.001**	**− 12.19 (− 17.14; − 7.23), *p* < 0.001**	**− 0.29 (− 0.39; − 0.19),** ***p* < 0.001**	**− 0.23 (− 0.32; − 0.13),** ***p* < 0.001**
Group 5 Frailty, < 3 comorbidities	**− 8.97 (− 16.10; − 1.84), *p* = 0.01**	**− 8.72 (− 15.71; − 1.73), *p* = 0.014**	**− 0.19 (− 0.33; − 0.05),** ***p* = 0.007**	**− 0.17 (− 0.31; − 0.04),** ***p* = 0.01**
Group 6 Frailty, 3 + comorbidities	**− 12.57 (− 18.53; − 6.61), *p* < 0.001**	**− 10.19 (− 16.11; − 4.27), *p* = 0.001**	**− 0.26 (− 0.38; − 0.15),** ***p* < 0.001**	**− 0.19 (− 0.30; − 0.07),** ***p* = 0.001**

Model 1 is unadjusted; Model 2 is adjusted for sex, age, level of education, and living arrangement. Bold values indicate significant associations (*p* < 0.05).

EQ-5D, EuroQoL-5 Dimension; VAS = Visual Analogue Scale. Group 1 includes non-frail individuals with < 3 comorbidities, Group 2 includes non-frail individuals with ≥ 3 comorbidities, Group 3 includes pre-frail individuals with < 3 comorbidities, Group 4 includes pre-frail individuals with ≥ 3 comorbidities, Group 5 includes frail individuals with < 3 comorbidities, Group 6 includes frail individuals with ≥ 3 comorbidities.

Considering the individual domains of the EQ-5D-3L (Table 3), adjusted binary logistic regression models found that, compared with Group 1, those with no frailty but ≥ 3 comorbidities (Group 2) were more likely to report problems with mobility, while those with frailty but < 3 comorbidities (Group 5) were more likely to report problems with mental health. Individuals who were living with pre-frailty or frailty and ≥ 3 comorbidities, reported significantly greater problems in all EQ-5D-3L domains compared to the healthiest ones (Group 1).

**Table 3:** Binary logistic regression for the association between multimorbidity/frailty groups and moderate/extreme problems in EQ-5D dimensions reported by patients with atrial fibrillation.

EQ-5D domains	Odds ratio (95% Confidence Interval), p-value					
	Multimorbidity/frailty groups					
	Group 1 No frailty, < 3 comorbidities	Group 2 No frailty, ≥ 3 comorbidities	Group 3 Pre-frailty, < 3 comorbidities	Group 4 Pre-frailty, ≥ 3 comorbidities	Group 5 Frailty, < 3 comorbidities	Group 6 Frailty, ≥ 3 comorbidities
Mobility	Ref	**2.62 (1.35; 5.09), *p* = 0.004**	1.14 (0.63;2.06), *p* = 0.60	**3.17 (1.79;5.62), *p* < 0.001**	0.99 (0.44;2.23), *p* = 0.97	**2.28 (1.16;4.50), *p* = 0.02**
Self-care	Ref	2.12 (0.80; 5.58), *p* = 0.13	0.63 (0.23;1.74), *p* = 0.37	**3.13 (1.33;7.39), *p* = 0.01**	1.70 (0.53;5.46), *p* = 0.37	**3.20 (1.24;8.24), *p* = 0.02**
Usual activities	Ref	1.69 (0.89; 3.20), *p* = 0.11	1.14 (0.66;1.70), *p* = 0.65	**2.71 (1.56;4.69), *p* < 0.001**	1.50 (0.70;3.20), *p* = 0.30	**2.08 (1.08;4.01), *p* = 0.03**
Pain/discomfort	Ref	1.85 (0.98; 3.47), *p* = 0.06	1.20 (0.70;2.05), *p* = 0.51	**2.13 (1.24;3.64), *p* = 0.01**	1.31 (0.62;2.76), *p* = 0.49	**2.12 (1.11;4.04), *p* = 0.02**
Mental health	Ref	1.79 (0.95; 3.38), *p* = 0.07	1.35 (0.78;2.33), *p* = 0.28	**2.39 (1.39;4.12), *p* = 0.002**	**2.26 (1.06;4.82), *p* = 0.04**	**2.00 (1.05;3.81), *p* = 0.04**

Models are adjusted for sex, age, level of education, and living arrangement. Bold values indicate significant associations (*p* < 0.05). Group 1 includes non-frail individuals with < 3 comorbidities, Group 2 includes non-frail individuals with ≥ 3 comorbidities, Group 3 includes pre-frail individuals with < 3 comorbidities, Group 4 includes pre-frail individuals with ≥ 3 comorbidities, Group 5 includes frail individuals with < 3 comorbidities, Group 6 includes frail individuals with ≥ 3 comorbidities.

### Problems in managing health

**Fig. 1:**
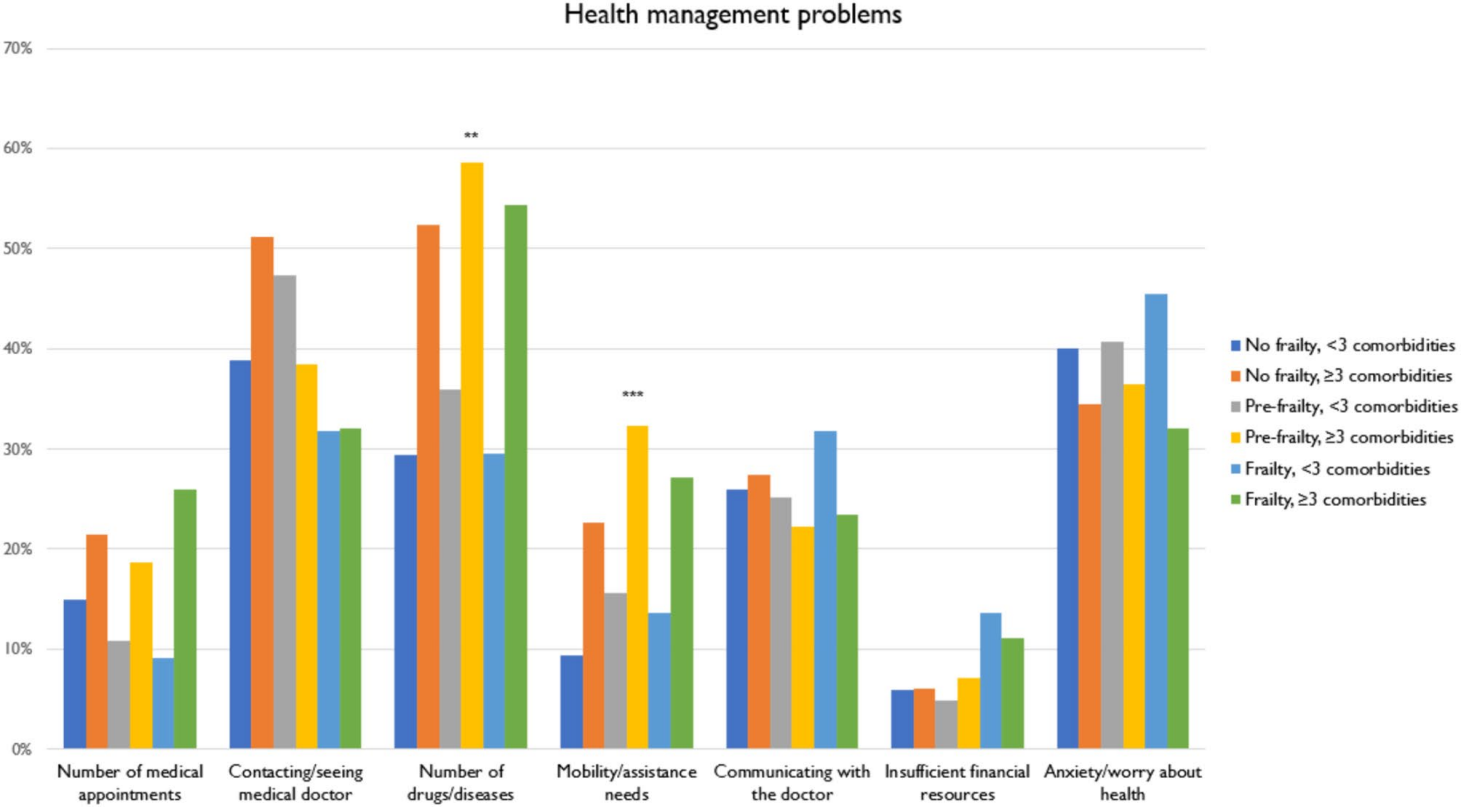
Frequencies of major health management problems by multimorbidity/frailty group. *Notes*. Group 1 includes non-frail individuals with < 3 comorbidities, Group 2 includes non-frail individuals with ≥ 3 comorbidities, Group 3 includes pre-frail individuals with < 3 comorbidities, Group 4 includes pre-frail individuals with ≥ 3 comorbidities, Group 5 includes frail individuals with < 3 comorbidities, Group 6 includes frail individuals with ≥ 3 comorbidities. *p < 0.05, **p < 0.01, ***p < 0.001.

The frequency of the main perceived health management problems by each group are presented in Fig. 1. For all groups, contacting or seeing a doctor, number of drugs and diseases, and anxiety/worry over their health were the most prevalent concerns, regardless of frailty or comorbidity group. A clear pattern emerges, with individuals in Groups 2, 4, and 6 (those with ≥ 3 comorbidities) more frequently reporting a higher number of medical appointments, a greater burden of chronic conditions, and increased need for assistance and mobility, especially when pre-frailty and frailty coexisted. Groups 5 and 6 were less concerned by the difficulty in seeing or contacting a doctor.

Binary logistic regression models (Supplementary Table S2 online) revealed a significant association between the number of chronic conditions, regardless of frailty status, and the probability of presenting some health management problems. Patients who were pre-frail or frail with ≥ 3 comorbidities were more likely to report problems with mobility and the need for assistance. Only individuals with no frailty or pre-frailty and ≥ 3 comorbidities (rather than those with frailty) were more likely to report the burden of managing multiple health conditions as a major issue. No significant associations in problems managing their health were found for patients in Groups 3 and 5, both characterized by having < 3 comorbidities, regardless of their frailty status.

### Important health outcomes

**Fig. 2:**
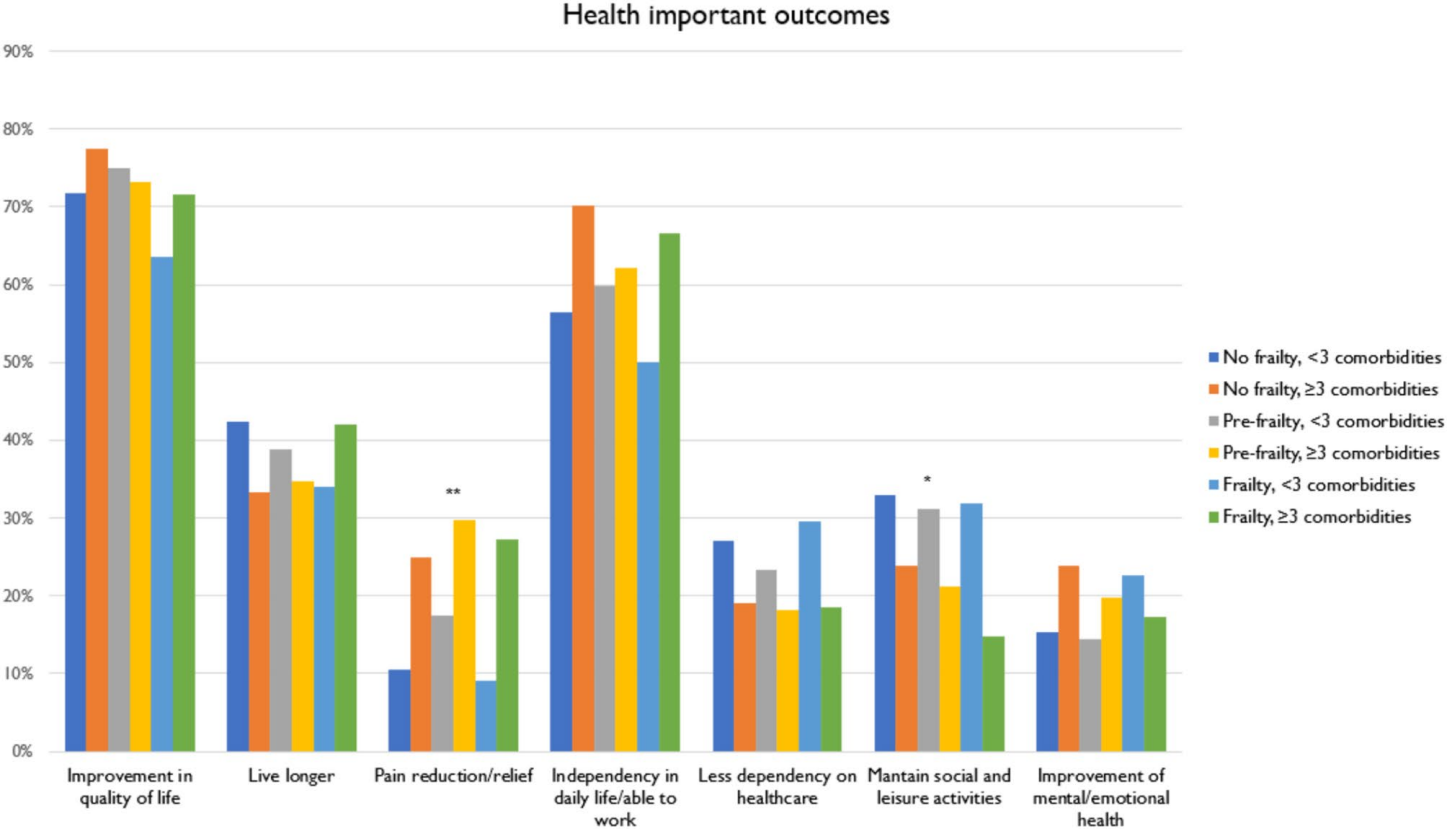
Frequencies for main health important outcomes by multimorbidity/frailty group. *Notes*. Group 1 includes non-frail individuals with < 3 comorbidities, Group 2 includes non-frail individuals with ≥ 3 comorbidities, Group 3 includes pre-frail individuals with < 3 comorbidities, Group 4 includes pre-frail individuals with ≥ 3 comorbidities, Group 5 includes frail individuals with < 3 comorbidities, Group 6 includes frail individuals with ≥ 3 comorbidities. *p < 0.05, **p < 0.01, ***p < 0.001.

Figure 2 represents the frequency of the main perceived health important outcomes by group. Improvements in QoL, maintaining independence in daily life and the ability to work emerged as the most frequently reported outcomes across all groups. These priorities were particularly emphasized by those with a higher number of comorbidities (Group 2, 4, and 6), in particular pain reduction or relief. Maintaining social and leisure activities and reducing dependency on healthcare were prioritized by those with less comorbidities (Group 1, 3, and 5). Conversely, frail and multimorbid individuals (Group 6) systematically placed less importance on maintaining social and leisure activities. Binary logistic regression models adjusted for potential confounders (Supplementary Table S3 online) confirmed a significant positive association between pain reduction/relief for all groups with 3 or more comorbidities (Groups 2, 4, and 6).

## Discussion

This study highlights the distinct and combined contributions comorbidity and frailty in shaping HRQoL, health management challenges, and patient-prioritized outcomes in older adults with AF. All groups, compared to those with no frailty and fewer comorbidities, reported a poorer perception of their health. The most pronounced impairments were observed in individuals with both pre-frailty or frailty and ≥ 3 comorbidities, highlighting the cumulative burden of these conditions. Importantly, a comparable reduction in HRQoL was observed in individuals with isolated frailty and those with isolated multimorbidity, suggesting that each condition independently exerts distinct yet similarly detrimental effects on perceived health. When co-occurring, their combined presence was associated with a markedly greater reduction in HRQoL, suggesting that their coexistence amplifies the overall burden beyond that observed with either condition alone. Frailty and comorbidity likely impact HRQoL through multiple pathways. Biologically, chronic inflammation and sarcopenia—hallmarks of frailty—may exacerbate fatigue and mobility limitations. Socially, frequent hospitalisations, fragmented care, and social isolation further deteriorate perceived health status.

In this cohort, frailty prevalence was 19% and pre-frailty 55.4%, consistent with prior reports. For example, a recent study of 2369 patients with AF reported frailty and pre-frailty prevalence of 10.6% and 60.7%, respectively^21^. However, the estimated prevalence of frailty in older adults with AF varies widely in the literature, ranging from 4.4 to 75.4%^22,23^, depending on sample populations and definitions of frailty employed^9^. Furthermore, available data on frailty prevalence in AF patients remains limited, emphasizing the need for further research^9^. Regarding comorbidity, our data aligns with a previous retrospective population study on individuals with AF that considered various criteria for defining comorbidity^24^, as reflected in the similar median number of chronic conditions (three) observed in our sample, further reinforcing the relevance of multimorbidity in this patient population.

Our findings show a clear gradient in QoL across the six groups, with non-frail and pre-frail individuals with less comorbidities reporting better HRQoL, while pre-frail and frail individuals with more comorbidities exhibited the lowest HRQoL. Notably, pre-frail individuals with less comorbidities showed HRQoL profiles comparable to those of the non-frail with less comorbidities, suggesting that pre-frailty alone, in the absence of comorbidity, may not substantially impair health perceptions or daily functioning in patients with AF.

These results align with longitudinal evidence indicating that pre-frailty represents a dynamic and potentially reversible state, with a proportion of individuals transitioning back to robustness over time^25^. Importantly, individuals who maintain or improve their frailty status over time exhibit significantly better physical and social quality of life compared with those whose frailty worsens^26^. Therefore, pre-frailty may represent an intermediate condition in which residual functional reserve, compensatory mechanisms and psychosocial resources are still sufficient to preserve perceived health status, underscoring the importance of early identification for timely, preventive interventions aimed at preserving HRQoL and delaying functional decline.

When examining specific HRQoL domains, notable distinctions emerged. Among non-frail individuals with ≥ 3 comorbidities, mobility problems emerged as the only significant challenge and were consistently reported across all groups with more comorbidities, reflecting the functional decline and physical impairments frequently associated with the presence of multiple chronic conditions. Beyond mobility,individuals with more comorbidities, regardless of frailty, more frequently reported difficulties in managing their health, with an increased burden related to medical appointments, polypharmacy, and mobility limitations. In contrast, among individuals with frailty but less comorbidities the only significantly impacted HRQoL domain was mental health. This pattern indicates that, in the absence of substantial disease burden, frailty may be particularly associated with psychological distress and perceived vulnerability. This group also had the highest proportion of individuals with higher educational attainment, which could represent a protective factor in the effective management of chronic conditions despite frailty, through mechanisms such as enhanced health literacy and improved socioeconomic status, ultimately reducing the risk of multimorbidity^27^. Higher education is generally linked to better health literacy and disease management^28^, yet in our sample, it was associated with a higher likelihood of reporting mental health concerns. This may reflect increased self-awareness of functional decline, though further research is needed to confirm this hypothesis.

The challenges reported by participants aligned closely with their clinical profiles. Individuals with more comorbidities, irrespective of frailty status, consistently identified the burden of managing multiple medications and health conditions as significant issues. These difficulties are exacerbated by fragmented care, requiring frequent medical appointments, consultations with multiple specialists, and navigating often poorly coordinated treatment plans. Additionally, polypharmacy not only demands strict adherence to complex medication regimens but also increases the risk of adverse drug interactions, further complicating symptom control and overall health management. These findings indicate that multimorbidity primarily translates into a tangible management burden and greater healthcare complexity.

Important differences emerged when considering frailty status. Individuals with pre-frailty and frailty and a higher number of comorbidities reported greater need for assistance and mobility-related challenges, reflecting the loss of independence. Conversely, individuals with more comorbidities but no frailty predominantly highlighted the difficulty of managing their conditions independently, suggesting that their autonomy shifts the burden of care management to themselves rather than caregivers or external support systems. Thus, while multimorbidity alone appears to increase management complexity, its coexistence with frailty is associated with greater functional dependency.

The prioritisation of outcomes aligned with these insights. Across all groups, improving QoL, maintaining independence and active engagement in occupational and daily activities were consistently ranked as the most important goals. However, some interesting group-differences emerged. Pain reduction was particularly emphasized by all individuals with ≥ 3 chronic conditions, consistent with the prevalence of pain-related conditions in our sample. Indeed, 12% of participants reported chronic pain, and approximately a quarter of them had osteoarthritis, a condition commonly associated with pain symptomatology. The association between multimorbidity and pain has been linked to adverse outcomes such as psychological distress, sleep disturbances, and increased healthcare utilisation^29^. Addressing pain management in these populations is therefore essential to mitigate its cascading effects on overall health.

Individuals with worse health status (frailty and more comorbidities) placed less importance on maintaining social and leisure activities. This may reflect not only a resignation to the limitations imposed by their conditions but also a shift in priorities, as the increasing burden of frailty and comorbidities leads them to focus more on immediate health concerns, such as pain relief. This finding illustrates the profound social impact of frailty and comorbidities, which not only affect physical independence but also reduce participation in meaningful activities, further diminishing QoL^13,14,30^.

Taken together, these findings suggest that frailty and comorbidity are related but non-redundant dimensions of vulnerability in patients with AF. A comparable reduction in HRQoL was observed among individuals with high comorbidity burden in the absence of frailty and those who were frail despite having fewer chronic conditions, underscoring that each condition, even when present in isolation, carries clinically meaningful consequences. Moreover, while comorbidity appears to be primarily linked to physical symptom burden and increased healthcare management complexity, frailty alone may be more closely associated with psychological vulnerability and mental health concerns. Their coexistence identifies individuals experiencing compounded impairment across both physical and psychosocial domains. This distinction may reflect differences in underlying pathophysiological mechanisms, whereby multimorbidity predominantly manifests through organ-specific symptom burden, whereas frailty reflects systemic vulnerability, reduced physiological reserve, and increased susceptibility to stressors.

Although our findings show a clear association between frailty, comorbidity and poorer HRQoL, the cross-sectional design of the study does not allow conclusions regarding causality to be made. While frailty and comorbidity plausibly contribute to reduced HRQoL, the opposite pathway is equally possible. Poor HRQoL, through mechanisms such as physical inactivity, depression, and reduced healthcare engagement, may contribute to frailty progression, as well as to the worsening or accumulation of chronic conditions over time^25,31^. These relationships are likely bidirectional and may evolve dynamically. Longitudinal studies are therefore needed to disentangle temporal trajectories and better understand how these dimensions interact and influence each other in aging in patients with AF.

Beyond the main findings, strengths and limitations of this study should be considered. One notable strength is the use of validated tools to assess frailty and QoL, ensuring the reliability and comparability of our results. Moreover, the inclusion of participants from multiple European countries provides a broader perspective on health challenges in individuals with AF, contributing to a more comprehensive understanding of frailty and comorbidity across different healthcare systems. However, some limitations should be acknowledged. Our study’s online survey format may have led to a selection bias, potentially underrepresenting the oldest (i.e., ≥ 85 years) individuals and those with more severe frailty and comorbidity burden. This may have resulted in an underestimation of the association between frailty, comorbidity and HRQoL levels. Indeed, it is plausible that this category of individuals would have reported even lower HRQoL and expressed greater need for assistance, mobility limitations, pain management, and loss of independence, alongside a further shift away from social and leisure activities toward more immediate and health-related concerns. Additionally, although models were adjusted for major sociodemographic factors, broader indicators of socioeconomic status and healthcare access, which may influence HRQoL disparities, were not measured. A further methodological consideration concerns the choice to use the EQ-5D-3L rather than the more recent 5L version, which was developed to enhance sensitivity and measurement precision. The EQ-5D-3L was used to reduce cognitive burden and overall questionnaire burden in an older, comorbid population, to balance comprehensiveness and feasibility while minimizing the risk of respondent fatigue and drop-out. Moreover, comorbidity was operationalised as a simple disease count, with conditions weighted equally and without accounting for differences in type, severity, or functional impact. While specific diseases and their clinical severity may have heterogeneous implications, the primary objective was to evaluate the cumulative burden of chronic conditions and its interplay with frailty, rather than to disentangle the specific contribution of individual conditions.

Finally, information on AF subtype (i.e., paroxysmal, persistent, or permanent) was not available. Given that different AF patterns may be associated with heterogeneous symptom burden, therapeutic strategies, and clinical trajectories, the absence of this information limited our ability to fully interpret differences in HRQoL and patient priorities. Future research should incorporate AF subtype stratification, longitudinal designs, and more diverse sampling strategies are warranted to better clarify these relationships.

### Implications for practice and future research

Our findings suggest that frailty and multimorbidity represent related but non-redundant dimensions of vulnerability in older adults with AF, with partially distinct implications for clinical care.

Assessing comorbidity burden alone may capture treatment workload and symptom-related challenges, but risks underestimating functional decline and psychosocial vulnerability. Conversely, focusing exclusively on frailty may overlook the cumulative impact of multiple chronic conditions on care complexity and pain-related outcomes. Integrating both dimensions into routine assessment may therefore refine risk stratification and support more differentiated care pathways.

Moreover, the identification of needs and primary challenges in this population underscore the need for coordinated, multidisciplinary care aimed at reducing treatment fragmentation and polypharmacy risks. Enhancing patient independence through accessible healthcare services, and community-based programs may reduce caregiver burden and improve continuity of care. Healthcare policies should integrate AF care with geriatric and primary care services, support non-pharmacological interventions such as exercise-based frailty management, and incorporating frailty assessment in international AF guidelines to optimise treatment decisions in older adults. Lastly, given the psychological impact of functional decline, routine screening for psychological distress, along with interventions that foster resilience and social engagement, should be standard components of care.

## Conclusions

In conclusion, frailty and comorbidity contribute to HRQoL in older adults with AF through partially distinct yet overlapping pathways, underscoring that they are crucial dimensions of vulnerability. Stratifying patients according to frailty and comorbidity status may help delineate clinically meaningful vulnerability profiles, capturing not only individuals with isolated frailty or comorbidity—who nonetheless experience comparable reductions in HRQoL—but also those in whom both conditions converge. Integrating this stratification into routine practice may support more nuanced, patient-centred decision-making, and ultimately improveQoL, mental well-being, patient independence, and the strain on caregivers^32^. Future research should further explore targeted interventions that enhance coordination between healthcare providers, address psychological and social determinants of health, and tailor treatments to the complex realities faced by this vulnerable population.

## Supplementary Information

Supplementary Information.

## Data Availability

The datasets generated during and/or analysed during the current study are not publicly available due privacy reasons but are available from the corresponding author on reasonable request.

## Abbreviations

AF: Atrial fibrillation
CI: Confidence Interval
COPD: Chronic obstructive pulmonary disease
EQ-5D-3L: EuroQol 5-Dimension, 3-level version
HRQoL: Health-related quality of life
IQR: Interquartile range
NH: Nursing home
QoL: Quality of life
VAS: Visual Analogue Scale
