# Supplementary material for: The role of comorbidity and frailty in shaping the burden of atrial fibrillation: a multinational cross-sectional survey
Source: Sci Rep. 2026 Mar 30;16:10562. doi: 10.1038/s41598-026-44800-1 (PMC13039896; doi:10.1038/s41598-026-44800-1)
Supplement: Supplementary file 1 — Supplementary Information. [file 41598_2026_44800_MOESM1_ESM.docx]

**SUPPLEMENTARY TABLES**

**Supplementary Table S1.** Main information collected in the AFFIRMO survey

| Main areas of interest investigated | Collected information |
| --- | --- |
| Sociodemographic characteristics | age, biological sex, level of education, living arrangements (living at home alone, living at home with family, living at home with part-time assistance, living at home with full-time assistance or living in a nursing home), marital status and smoking habits |
| Medical history (comorbidities) | arterial hypertension, heart diseases, thyroid problems, chronic obstructive pulmonary disease (COPD), diabetes mellitus, gastrointestinal diseases, chronic kidney diseases, chronic liver disease, Parkinson's disease, previous stroke, multiple sclerosis, cognitive disturbances, osteoporosis, osteoarthritis, rheumatoid arthritis, oncologic diseases, chronic pain, and sensory (vision and hearing) deficits. |
| Health management problems | number of medical appointments, having too many medications to take, having too many health problems to manage, not having enough financial resources, difficulties in contacting/seeing a medical doctor, anxiety/worry of own health, mobility/assistance needs, do not understand medical recommendations clearly, not having the opportunity to talk freely with the doctor about doubts and fears about the health conditions and treatment. |
| Health outcomes of importance | improve quality of life, live longer, pain reduction/relief, be independent in daily life/be able to work, have less need for health care, maintain social and leisure activities, and improve mental and emotional health. |

**Supplementary Table S2.** Binary logistic regression for the association between comorbidity/frailty groups and main problems in managing health reported by patients with atrial fibrillation.

| **Main problems in managing health** | **Odds ratio (95% Confidence Interval), p-value** | | | | | |
| --- | --- | --- | --- | --- | --- | --- |
|  | **Multimorbidity/frailty** **Groups** | | | | | |
|  | **Group 1**  **No frailty,**  **<3 comorbidities** | **Group 2**  **No frailty,**  **≥3 comorbidities** | **Group 3**  **Pre-frailty,**  **<3 comorbidities** | **Group 4**  **Pre-frailty,**  **≥3 comorbidities** | **Group 5**  **Frailty,**  **<3 comorbidities** | **Group 6**  **Frailty,**  **≥3 comorbidities** |
| Number of medical appointments | Ref. | 1.34 (0.59;3.02), p=0.49 | 0.69 (0.31;1.49), p=0.34 | 1.12 (0.55;2.30), p=0.76 | 0.59 (0.18;1.98), p=0.40 | 1.70 (0.76;3.80), p=0.20 |
| Contacting/seeing medical doctor | Ref. | 1.78 (0.95;3.32), p=0.07 | 1.41 (0.82;2.41), p=0.21 | 1.09 (0.64;1.86), p=0.76 | 0.75 (0.35;1.65), p=0.48 | 0.85 (0.44;1.64), p=0.64 |
| Number of drugs/diseases | Ref. | **2.73 (1.43;5.20), p=0.002** | 1.35 (0.76;2.38), p=0.31 | **3.42 (1.95;6.00), p<0.001** | 0.99 (0.44;2.23), p=0.99 | **2.69 (1.40;5.19), p=0.003** |
| Mobility/assistance needs | Ref. | 2.25 (0.90;5.59), p=0.08 | 1.84 (0.78;4.33), p=0.16 | **3.82 (1.71;8.57), p=0.001** | 1.44 (0.46;4.53), p=0.53 | **2.86 (1.16;7.08), p=0.02** |
| Communicating with the doctor | Ref. | 1.19 (0.59;2.41), p=0.63 | 1.01 (0.54;1.86), p=0.99 | 0.93 (0.51;1.72), p=0.82 | 1.45 (0.64;3.30), p=0.38 | 1.07 (0.51;2.23), p=0.85 |
| Insufficient financial resources | Ref. | 1.15 (0.31;4.24), p=0.84 | 0.84 (0.26;2.72), p=0.77 | 1.30 (0.44;3.8), p=0.63 | 2.63 (0.73;9.52), p=0.14 | 2.12 (0.65;6.93), p=0.22 |
| Anxiety/worry about health | Ref. | 0.95 (0.50;1.81), p=0.89 | 1.04 (0.61;1.80), p=0.88 | 1.01 (0.59;1.74), p=0.96 | 1.39 (0.66;2.95), p=0.39 | 0.88 (0.46;1.70), p=0.71 |
| Having lots of health problems | Ref. | **3.30 (1.41;7.70), p=0.006** | 1.07 (0.46;2.51), p=0.88 | **4.32 (2.01;9.27), p<0.001** | 0.58 (0.15;2.28), p=0.43 | 2.17 (0.90;5.26), p=0.09 |
| Having too medications to take | Ref. | **2.15 (1.05;4.38), p=0.04** | 1.40 (0.74;2.65), p=0.31 | **1.87 (1.00;3.50), p=0.05** | 1.31 (0.55;3.15), p=0.54 | **2.60 (1.27;5.30), p=0.01** |

*Notes*: All models were adjusted for sex, age, level of education, and living arrangement. Group 1 includes individuals non-frail individuals with <3 comorbidities, Group 2 includes non-frail individuals with ≥3 comorbidities, Group 3 includes pre-frail individuals with <3 comorbidities, Group 4 includes pre-frail individuals with ≥ 3 comorbidities, Group 5 includes frail individuals with < 3 comorbidities, Group 6 includes frail individuals with ≥ 3 comorbidities.

**Supplementary Table S3.** Binary logistic regression for the association between comorbidity and frailty groups and health outcomes important to patients with atrial fibrillation.

| **Health outcomes** | **Odds ratio (95% Confidence Interval), p-value** | | | | | |
| --- | --- | --- | --- | --- | --- | --- |
|  | **Multimorbidity/frailty Groups** | | | | | |
|  | **Group 1**  **No frailty,**  **<3 comorbidities** | **Group 2**  **No frailty,**  **≥3 comorbidities** | **Group 3**  **Pre-frailty,**  **<3 comorbidities** | **Group 4**  **Pre-frailty,**  **≥3 comorbidities** | **Group 5**  **Frailty,**  **<3 comorbidities** | **Group 6**  **Frailty,**  **≥3 comorbidities** |
| Improvement in quality of life | Ref. | 1.74 (0.85;3.57), p=0.13 | 1.14 (0.62;2.08), p=0.68 | 1.34 (0.74;2.42), p=0.34 | 0.69 (0.31;1.52), p=0.36 | 1.28 (0.63;2.60), p=0.50 |
| Live longer | Ref. | 0.70 (0.37;1.32), p=0.27 | 0.84 (0.49;1.43), p=0.51 | 0.73 (0.43;1.25), p=0.25 | 0.68 (0.31;1.46), p=0.32 | 0.99 (0.53;1.87), p=0.98 |
| Pain reduction/relief | Ref. | **2.71 (1.14;6.39), p=0.02** | 1.84 (0.82;4.11), p=0.14 | **3.41 (1.58;7.35), p=0.002** | 0.83 (0.24;2.88), p=0.77 | **2.91 (1.23;6.90), p=0.02** |
| Independency in daily life/able to work | Ref. | 1.76 (0.92;3.37), p=0.09 | 1.21 (0.71;2.07), p=0.48 | 1.24 (0.73;2.1), p=0.43 | 0.81 (0.39;1.71), p=0.59 | 1.55 (0.81;2.95), p=0.19 |
| Less dependency on health care | Ref. | 0.58 (0.28;1.22), p=0.16 | 0.86 (0.47;1.58), p=0.63 | 0.57 (0.31;1.06), p=0.08 | 1.17 (0.51;2.6), p=0.71 | 0.59 (0.28;1.27), p=0.18 |
| Maintain social and leisure activities | Ref. | 0.68 (0.3;1.37), p=0.28 | 0.93 (0.52;1.6), p=0.81 | 0.61 (0.34;0.11), p=0.11 | 0.90 (0.40;2.02), p=0.80 | **0.42 (0.19;0.94), p=0.03** |
| Improve mental/ emotional health | Ref. | 2.01 (0.91;4.46), p=0.08 | 0.94 (0.45;1.98), p=0.87 | 1.54 (0.76;3.13), p=0.23 | 1.76 (0.69;4.51), p=0.23 | 1.43 (0.61;3.33), p=0.41 |

*Notes*: All models were adjusted for sex, age, level of education, and living arrangements. Group 1 includes individuals non-frail individuals with <3 comorbidities, Group 2 includes non-frail individuals with ≥3 comorbidities, Group 3 includes pre-frail individuals with <3 comorbidities, Group 4 includes pre-frail individuals with ≥ 3 comorbidities, Group 5 includes frail individuals with < 3 comorbidities, Group 6 includes frail individuals with ≥ 3 comorbidities
